# Comment on “Bladder Cancer–Specific Nuclear Matrix Proteins-4 May Be a Potential Biomarker for Non-Muscle-Invasive Bladder Cancer Detection”

**DOI:** 10.1155/2019/6432596

**Published:** 2019-11-21

**Authors:** Gökçe Güllü Amuran

**Affiliations:** Marmara University Faculty of Medicine, Istanbul, Turkey

I have read the article “Bladder Cancer Specific Nuclear Matrix Proteins-4 May Be a Potential Biomarker for Non-Muscle-Invasive Bladder Cancer Detection” with great interest [1]. The article reports that the urine concentration of BLCA-4 protein is a strong candidate for the diagnosis of non-muscle-invasive bladder cancer [1]. However, no normalization strategy was used to determine urinary protein concentrations. In order to eliminate physiological differences such as dehydrated individuals, urinary protein concentrations should be normalized according to proteins such as creatinine [2]. If normalization is done, I would kindly ask you to share data on normalization. If normalization is not done, I would like you to share information about how physiological condition differences between individuals are excluded.
